# Strategies deemed important by frontline healthcare providers for their mental wellbeing during first wave of COVID-19 pandemic

**DOI:** 10.1007/s44202-022-00028-4

**Published:** 2022-02-15

**Authors:** Majid Alabdulla, Ovais Wadoo, Yousaf Iqbal, Nirvana Swamy Kudlur Chandrappa, Prem Chandra, Naseer Ahmed Masoodi, Muna A. Rahman S. Al-Maslamani, Javed Latoo

**Affiliations:** 1 Hamad Medical Corporation, Doha, Qatar; 2 Qatar University, Doha, Qatar

**Keywords:** COVID-19, Healthcare workers, Infection control, Mental wellbeing, Qatar

## Abstract

**Background:**

Global research so far has demonstrated a significant psychological impact on frontline healthcare workers and the need to support them. Mitigation strategies are vital to ensure psychological wellbeing of healthcare workers and should take healthcare workers experiences and views into consideration. However, qualitative research on this topic has been limited from the Arab world and we set out to fill this research gap. The objective was to understand participants’ emotional experiences and identify their valued aspects of support, to inform potential mitigation strategies for their psychological wellbeing.

**Methods:**

Content analysis of free-text comments of a web-based survey of healthcare workers associated with the COVID-19 designated hospital and quarantine sites in Qatar, during the first wave of COVID-19 pandemic. Extracts and phrases were used to identify potential themes, with relevant quotes gathered within identified themes.

**Results:**

A total of 779 staff members were invited to participate in this study and 286 responded. The results indicate that strategies around infection control practice, personal protective equipment, SARS-CoV-2 testing, workload, financial compensation, religion, psychological support and engaged leadership are deemed important by frontline healthcare workers to maintain their mental wellbeing.

**Conclusions:**

Mitigating factors identified by healthcare workers to protect their psychological wellbeing should inform the organizational strategy. Resources which enhance mental wellbeing should be easily and consistently available to all staff.

## Background

Coronavirus disease 2019 (COVID-19) is an infection with severe acute respiratory syndrome coronavirus 2 (SARS-CoV-2). The illness due to this virus ranges from mild symptoms to severe illness and death. By January 2020, World Health Organization (WHO) declared it as a Public Health Emergency of International Concern and by March 2020 it was characterized as a pandemic. Global research so far has demonstrated a significant psychological impact on frontline healthcare workers and the need to support them [1–3]. Mental health problems commonly include symptoms of anxiety, depression, trauma and insomnia which can impact the functioning of healthcare workers. Mitigation strategies were introduced by policymakers to enhance the mental health and resilience of frontline healthcare workers which included interventions to support basic daily needs, psychological support interventions, and pharmacological interventions [4]. Mitigation strategies should take healthcare workers experiences and views into consideration and these views can be ascertained by qualitative studies. However qualitative research has been limited during infectious epidemics as it is deemed too intrusive or burdensome for research participants [5]. A recent meta-synthesis of the qualitative studies by Billing and colleagues [6] identified 46 studies which explored healthcare workers’ experiences and views from pandemics or epidemics including and prior to COVID-19. The meta-synthesis included 15 studies exploring experiences of healthcare workers during Ebola, 14 during SARS, five during COVID-19, four during unspecified influenza pandemics, three during MERS, three during H1N1,one during both SARS and H1N1, one during Avian flu, one during swine flu and one during general public health emergencies. The main theme that emerged from these studies was the anxiety about physical safety, especially in the early phases of epidemics. Inadequate Personal Protective Equipment (PPE), insufficient resources, high workloads, long shifts, stigma and inconsistent information have been identified as factors exacerbating the anxiety. Of importance to note is, that only one study in this meta-synthesis pertains to COVID-19 pandemic in the Arab world (Lebanon) [7]. Of the studies from previous epidemics, only one study was carried out in the Arab world (KSA) [8]. The aim of the Lebanese study was to examine the psychosocial effects of being quarantined following exposure to COVID-19 among Lebanese health care workers. 13 health care workers participated in semi-structured interviews out at various COVID-19 units. This study revealed four themes namely, fear of contracting and spreading the virus, conflict between professional duty and family obligation, stigma of being infected and inadequate or inaccurate information. The other study from KSA was based on focus group interviews with 28 participants of whom nine were management decision-makers and 19 frontline healthcare workers. This study examined stakeholder perspectives of the factors contributing to or detracting from successful infection control management of a serious Middle East Respiratory Syndrome caused by a coronavirus (MERS-CoV) outbreak.

Qualitative research to inform the mitigation strategies has been limited in the Arab world and an important research gap. To our knowledge, ours is the first study in Qatar and the Arab world which attempts to not only understand participants’ emotional experiences but also to identify any support/intervention they deem valuable for their psychological wellbeing. We set out to fill this research gap by performing content analysis of free-text comments. Our aim was to inform areas of potential mitigation strategies and workplace interventions in the Arab world, which has its unique demographic and sociocultural context.

## Materials and methods

### Study design and participants

In this study, we set out to perform content analysis of free-text comments of a web-based survey. Surveys often include open-ended questions to allow participants to provide additional information. The responses to these free-text comments are often not evaluated and the sole reliance on numerical data has been criticized [8]. Evaluation of these open-ended questions can provide deeper insight into the data. Free-text comments were analyzed from two questions:

1. “Write your mental health feelings (e.g. anxious, fearful, irritable, low etc.) and experiences (e.g. impaired sleep, concentration or any other relevant experience) while working in hospital caring for patients with suspected or confirmed COVID-19 patients”
2. “Write what will help you to manage your mental wellbeing during these COVID-19 crises so that you can function well to continue caring for patients.”

This study was part of a larger project; a cross-sectional, web-based survey of healthcare workers managing suspected or confirmed COVID-19 patients in Qatar in the months of April and May 2020 [9, 10]. All healthcare workers associated with the COVID-19 designated hospital and quarantine sites (779) were invited through an official email and 36.7% responded. The primary outcome of interest in the study was mental wellbeing as measured by the Warwick-Edinburgh Mental Well-Being Scale (WEMWBS). The study collected demographic data, measures of mental wellbeing and free text comments from health care workers.

### Ethical approval

The study was granted ethical approval by the Medical Research Council of the Hamad Medical Corporation.

## Results

### Participants characteristics

A total of 190 (66.4%) of participants were male. Of all the participants, 203 (68.1%) were less than 35 years old, 20.9% were aged 35–44 and 7.9% were aged 45–65. The participants included 139 (48.6%) physicians, 115 (40.2%) nurses and 32 (11.1%) allied healthcare workers. The participants were from diverse ethnic backgrounds with majority being Arabs 87 (30.3%), Indian 74 (25.8%) and Philippine 66 (23.0%) (see Table 1).

**Table 1:** Participants characteristics

Characteristics	Frequency	Percentage (%)
Age group (in years)		
18–24	8	2.7
25–34	195	68.1
35–44	60	20.9
45–54	18	6.2
55–65	5	1.7
Gender		
Male	190	66.4
Female	94	32.8
Unknown	2	0.6
Ethnicity		
Arab-Qatari	2	0.6
Arab-Other	85	29.7
Indian	74	25.8
Pakistani	16	5.5
Philippines	66	23.0
African	27	9.4
Others	13	4.5
Unknown	3	1.0
Profession		
Doctor	139	48.6
Nurse	115	40.2
Allied healthcare workers	32	11.1

### Content analysis

Content analysis is a research tool used to determine the presence of certain words or concepts within texts or sets of texts. Researchers quantify and analyze the presence, meanings and relationships of such words and concepts, then make inferences about the messages within the texts or sets of texts [11, 12]. Free text comments were analyzed to enable the identification and generation of initial codes and textual units for features and patterns in the data. Extracts and phrases were used to identify potential themes, with relevant ‘quotes’ gathered within identified themes. Initial analysis was conducted by one of the authors and the data were systematically reviewed by two authors to ensure that a set of data were identified to support each theme. For trustworthiness reasons, peer debriefing was employed throughout the analysis to enhance credibility in keeping with best practice qualitative methodology.

### Themes and quotes: participants’ description of their emotions

See Table 2.

**Table 2:** Participants’ description of their emotions

Themes	Quotes
Anxiety and Depressive type symptoms	*…every time I step out of the house, I am praying not to bring back virus with me*
	*…anxious, fearful and irritable moments most of the days…because of that I am having impaired sleep (severe insomnia) and sometimes depression*
	*… feel anxious about contracting the disease at work. A bit impaired sleep due to shift work*
	*… was anxious ….. had impaired sleep which led to impaired concentration but most of all, I missed my family and my daughter too much*
	*feeling low and anxious, mostly due to current situation, uncertainty, different duty schedule and not being able to have quality time with family and friends*
	*…first few weeks handling COVID patients was a bit okay. But as days passed and number of cases started to increase, I feel a bit tired of the situation. Unfortunately, cancelling leave is something that added more to the stress of going to work with knowing you're not going to get vacation this year*
	*Feeling irritable with low self-esteem and anxiety*
	*Extremely stressful and feeling irritable*
Mixed anxiety and hope	*…impaired sleep and overwhelmed at home and at work but I do feel a sense of gratification and fulfillment knowing that I am trying the best I can to help fight this pandemic*
	*… consider myself lucky to play my part to fight off this pandemic under the kind supervision of our seniors. Sometimes I get headaches due to work burden but that’s no big deal*
	*…feeling fearful, worried but still optimistic*
	*… often feel relaxed, because I made the choice of being in this place, trying to give help during this global pandemic, my only issue is the sleep disturbance, and sometimes the continuous changing of policies*

### Themes and quotes: participants’ valued aspects and suggestion to maintain their mental wellbeing

See Table 3.

**Table 3:** Participants’ valued aspects and suggestion to maintain their mental wellbeing

Themes	Quotes
Infection control strategies	*…. peace of mind while I work that I have all protection for me (PPE) while I go see patients with suspected or confirmed corona case*
	*….as a staff nurse working directly with patients with corona virus, provision of enough and abundant PPE to protect ourselves at all times, even when staying at the nursing station is important*
	*… presence of Infection control Team 24* × *7 at each ward and Emergency Department areas to ensure compliance*
	*… need to be regularly tested too to have the confirmation that I am safe and sound around my family*
Engaged and empathetic leadership	*…the daily doctors’ morning meeting is helpful even for mental health. It makes me feel like I am not isolated and expressing and hearing the other doctors' concerns feels like a support group. I feel better when I attend the meeting whether online or physically* *…what helps is having emotional support from superiors and feeling that someone takes care of us as we take care of people*
	*…good supervisor, teamwork and clear guidelines will help so much* *…a supportive leader, someone could stand for us and who really cares for the front liners who are going through a fear of unknown is helpful*
	*…a positive and healthy attitude of administration and senior physicians towards the frontline workers is of utmost importance during this time of uncertainty. The insurance of our health in case we get exposed or sick is a mental relief*
	*…getting help and support from my managers and also getting recognition and appreciation for the biggest job we are doing during this crisis*
Work pattern, environment and financial compensation	*…reducing working hours and organizing off days helped much to make work easier and less stressful*
	*…flexibility in working hours, proper breaks and less paperwork will help*
	*…flexibility in decision making and more involvement of staff regardless of rank or job description. Recognition of efforts and financial compensation*
	*…adequate staffing at all levels specially physicians*
	*…more relaxed breaks and shorter working hours if possible*
	*…offering risk allowance and financial compensation for current work*
Religion and spirituality	*…praying to God and putting all affairs into His care*
	*…praying and Reading Quran*
	*…spirituality and meditation*
	*…belief in one God*
Psychological support	*…we nurses are definitely in need of a counselling session or some sort of encouragement to help us get going in our work*
	*…getting psychological and all other support from my teammates*
	*…online psychiatric consultation*
	*…counselling with family members and proper health care provision for the family as priority*
General measures	*…updated information about COVID 19,*
	*… have my family with me in this trying times. Knowing that I will go home and see them makes me come to my senses and function as I am expected. So long as they are okay, I will be okay*
	*…provision of a separate single accommodation for each staff living with family so they do not fear taking infection home from hospital*
	*…positive thinking: Being useful and being able to do a good job is what makes a person survive coronavirus pandemic. I can feel that I make a difference*

## Discussion

The results of our study indicate that the emotional experiences of frontline healthcare workers in our setting resonate with published literature. Healthcare workers at the frontline are at the greatest risk of contracting the disease. The fear of contracting the disease, uncertainty, rapid change and critically restructured priorities within the healthcare system are some of the factors that have been reported to increase anxiety [13, 14]. A recently published review of the mental health impact of working during epidemics and pandemics like SARS, MERS, Ebola and COVID-19 suggested that healthcare workers exposed to virus-related work are 1.7 times more likely to develop psychological distress and PTSD compared to non-exposed workers [15]. Some of the healthcare workers in our study derived meaning and satisfaction from their work as they were able to help others during this global pandemic. It is well known that most healthcare workers are inherently motivated and undertake work due to a sense of professional duty [6, 16]. The factors that our participants value and link to enhanced psychological wellbeing include adequate provision of Personal Protective Equipment (PPE), adherence to infection control practices, routine SARS-CoV-2 testing, shorter working hours, working in area of expertise, intermittent break from COVID roles and adequate financial compensation. This resonates with data from current and previous epidemics, that has identified need for better support for frontline workers such as provision of safety equipment, manageable workloads, relevant practical timely training issues, clear consistent communication, peer support systems, work family life balance and improved team working [17–23]. Compensation has been reported to be important by our participants, which is similar to what has been reported in Democratic Republic of the Congo during the Ebola hemorrhagic fever epidemic [24]. Similarly, a survey carried out in United Kingdom indicates that poor compensation and feeling under-valued were nurses’ primary concerns [25]. In addition to this, healthcare workers in Qatar value engaged, caring and supportive leadership, which reflects that they want more collaboration and consultation. Many operational difficulties stem from inequalities of power between management and front-line workers [26]. Healthcare workers desire clear and consistent information, and this appears to mitigate anxiety and stress [27–29]. Participants identified religion as an important aspect to maintain wellbeing. Religion and spirituality are known to offer a sense of connection with something bigger and helps people cope with difficult life situations. Self-coping styles and psychological growth plays an important role in maintaining mental health of nurses [30]. Peer support was identified as important for wellbeing in addition to specialist support.

## Strengths and limitations

This study contributed to the recent stream of qualitative research which attempts to not only understand participants’ emotional experiences but also to identify any support/intervention they deem valuable for their psychological wellbeing and is the first in Qatar and Arab world. Our research design, content analysis of free texts, enabled us to access a large set of qualitative data during a pandemic. By including an open-ended question in a cross-sectional, web-based survey, we were able to gain valuable additional information. The analysis was done as it complements quantitative data in creating mitigation strategies. However, one of the important limitations is lack of depth to responses, in comparison with interviews. Finally, our approach was mainly descriptive and exploratory, and the findings might not be generalizable to other countries with different health systems. Future studies could investigate this issue in more detail, using focus groups or interviews.

## Conclusion

This study provides a quick valuable qualitative analysis and feedback to those planning the support of frontline workers during this pandemic crisis. Our findings, though subject to limitations indicate that organizational factors like infection control practice, personal protective equipment, SARS-CoV-2 testing, workload and financial compensation should be addressed to prevent adverse mental health outcomes. Work environments that facilitate these basic practical and environmental needs lead to greater psychological well-being and work environments that frustrate these needs can cause psychological distress.
